# Platelet-to-lymphocyte ratio and serum hsCRP levels in third trimester and adverse pregnancy outcomes in women with gestational diabetes mellitus

**DOI:** 10.1038/s41598-023-48371-3

**Published:** 2023-11-28

**Authors:** Jun Tao, Yun Huang, Yan Li, Wen Dai

**Affiliations:** Department of Clinical Laboratory, Institute of Translational Medicine, Renmin Hospital of Wuhan University, Wuhan, China

**Keywords:** Immunology, Endocrinology

## Abstract

Gestational diabetes mellitus (GDM) is a major complication of pregnancy. GDM is associated with a higher risk of adverse pregnancy outcomes (APO). The purpose of this study was to assess the association between third-trimester platelet to lymphocyte ratio (PLR) and high-sensitivity C-reactive protein (hsCRP) concentration and the risk of APO in GDM pregnant women. This study selected 406 non-elderly gestational diabetes patients diagnosed in the Renmin Hospital of Wuhan University from May 2021 to February 2023 as the research objects. According to the presence or absence of APO, they were divided into an APO group (n = 171) and a non-APO group (n = 235). Logistic regression model to evaluate the correlation between PLR and hsCRP and APO in women with GDM; Restricted cubic spline analyses was used to explore nonlinear correlations between PLR or hsCRP and the risk of APO; ROC curve analysis of the diagnostic performance of PLR and hsCRP for APO in women with GDM. APO occurred in 171 of the 406 included participants. Compared with the non-APO group, patients in the APO group had higher PLR and hsCRP levels. The incidence of APO was positively associated with PLR and the hsCRP level in each logistic regression model (*P* < 0.05). After adjusting for all the risk factors included in this study, restricted cubic spline analyses found that the PLR and the hsCRP level were positively associated with the risk of APO. The levels of PLR and hsCRP in the third trimester are related to the occurrence of APO in women with GDM, and high levels of PLR and hsCRP may indicate the occurrence of APO.

## Introduction

Gestational diabetes mellitus (GDM) refers to a variable degree of abnormal glucose metabolism that occurs for the first time during pregnancy and is detected. It is the most common pregnancy-related metabolic disorder, occurring in about 7% of all pregnancies, and is usually detected in the second and third trimesters^1–3^. Women with a history of GDM had increased rates of metabolic syndrome and cardiovascular events compared with women without a history of GDM^4,5^. At the same time, studies have shown that about 10% of women develop diabetes soon after giving birth, and the rest have a 20–60% incidence of diabetes within 5–10 years in the absence of specific diabetes interventions^6,7^. GDM is associated with adverse pregnancy outcomes (APO), including preterm delivery, stillbirth, macrosomia, small for gestational age infant, and large for gestational age infant^8–10^.

The causes of APO in pregnant women with GDM are unclear, but may be related to insulin resistance (IR), genetic susceptibility, disorders of the insulin signaling system, decreased pancreatic islet B cell secretory function, chronic inflammation, various placental hormones (estradiol, cortisol, prolactin, progesterone) and various adipocytokines (TNF-α, leptin, adiponectin) in pregnant women are related to many factors^10–14^.

Both platelet-to-lymphocyte ratio (PLR) and high-sensitivity C-reactive protein (hsCRP) are commonly used indicators to reflect the inflammatory state of the body. Previous studies have shown that platelets release a variety of inflammatory mediators to participate in immune inflammatory responses through autocrine or paracrine methods, and at the same time directly chemotactic immune cells to aggregate and infiltrate into damaged tissues^13,15,16^. PLR has emerged as an informative marker, revealing changes in platelet and lymphocyte counts due to acute inflammation and prothrombotic states. Serum hsCRP, as an inflammatory mediator that rises sharply in plasma during inflammatory response, can play a regulatory role during inflammatory response by enhancing the phagocytic function of phagocytes and activating complement^17,18^. Numerous studies have confirmed that PLR and hsCRP are potential biomarkers for spontaneous preterm birth, miscarriage, and fetal growth restriction^19–23^. This study aimed to determine whether third trimester PLR and hsCRP are associated with APO in patients with GDM.

## Materials and methods

### Study population

We used a retrospective study design to evaluate the association between PLR and serum hsCRP levels and the risk of subsequent and APO in women with GDM. All participants were recruited consecutively between May 2021 and February 2023 at Renmin Hospital of Wuhan University in Obstetrics and Gynecology. All participants were diagnosed with GDM and under 35 years old. All women who were not diagnosed with diabetes before 24 weeks of gestation underwent a 75 g oral glucose tolerance test (OGTT) at 24–28 weeks of gestation. GDM was diagnosed using the criteria of the International Diabetes and Pregnancy Study Group (IADPSG)^24^, if one or more blood glucose values were equal to or exceed the defined cutoff values, namely, fasting blood glucose levels < 5.1 mmol/l and blood glucose levels of < 10.0 mmol/l, < 8.5 mmol/l during the first and second hours. The exclusion criteria were those patients (1) who presented with cardiogenic shock, heart valve disease; (2) who presented with uremia or have had kidney transplantation; (3) who suffer from severe liver disease, cancer, autoimmune diseases, blood system diseases, or infectious disease and (4) those who used anti-inflammatory drugs within four weeks.

The research protocol has been approved by the Ethics Committee of the Renmin Hospital of Wuhan University, and the research has been carried out in accordance with the Helsinki statement. Almost all patients or their relatives signed written informed consent before the study.

### Clinical assessments

Detailed medical histories and physical examination results were obtained from all included patients. The following baseline data from blood sample examinations were recorded from the first visit of the patients: age (years), gestational weeks, Pre-pregnancy BMI, maternal family history of diabetes, history of hypertension, preeclampsia, and the use of insulin. Pregnancy weight gain (kg) was counted after the end of pregnancy.

### Adverse pregnancy outcomes

Adverse pregnancy outcomes (including preterm delivery and/or stillbirth and/or macrosomia and/or postpartum hemorrhage and/or small for gestational age infant (SGA) and/or large for gestational age infant (LGA)) was used as the criteria to define cases.

### Blood sampling and analysis

At the time of the patient's admission, medical staff collected 5 ml of procoagulant blood and 2 ml of ethylenediaminetetraacetic acid (EDTA) anticoagulated venous whole blood from the cubital vein. The procoagulant blood was placed at room temperature for 30 min. After the blood coagulated, it was centrifuged at 3500 r/min (1 369 × g) for 5 min. The upper serum was collected in an Eppendorf tube and stored in the refrigerator at − 80 °C for later use. A Siemens Advia 2400 automatic biochemical analyser (Siemens, Erlangen, Germany) was used to determine serum Glu(glucose) and hsCRP. Johnson & Johnson Vitros 350 dry-biochemical analyzer (Johnson & Johnson, New Jersey, USA) was used to detect the urinary protein level. A SysmexCA-7000 system (Sysmex, Kobe, Japan) was used to detect the RBC (Red blood cell count), Hb(hemoglobin), RDW (Red blood cell distribution width), WBC (White blood cell count), NEU (Neutrophil count), LYM (lymphocyte count), MONO(monocyte count), PLT (Platelets), MPV (Mean platelet volume), PDW (Platelet distribution width).

### Statistical methods

Analyses were done with SPSS 23.0 and Python 3.9.7. PLT was the continuous variables that obeyed a normal distribution, so they are described by mean ± standard deviation. Age, gestational weeks, BMI, Glu, Urinary protein, RBC, Hb, RDW, WBC, NEU, LYM, MONO, MPV, PDW, NLR (Neutrophil–lymphocyte ratio), LMR (Lymphocyte-monocyte ratio), PLR, hsCRP, which did not obey the normal distribution and are expressed as interquartile range. The two-independent-sample t test or the Mann–Whitney U test were used to compare groups on the averages of continuous variables. The chi-square test t was used to compare the percentages of categorical variables between APO group and non-APO group. Simple and multiple logistic regression were used to explore the relationship between PLR and hsCRP and APO. Restricted cubic spline analyses were used to explore the nonlinear correlation between the PLR or the hsCRP and the risk of APO. ROC curve analysis of the diagnostic performance of PLR and hsCRP for APO in women with GDM.

### Ethics approval and consent to participate

The authors are accountable for all aspects of the work to ensure that issues related to the accuracy or completeness of any part of the work are properly investigated and resolved. This study was approved by the Medical Ethics Review Committee of Renmin Hospital of Wuhan University, China. All participants signed an informed consent form in accordance with the policies of the Renmin Hospital of Wuhan University Ethics Committee.

## Results

### Clinical characteristics

**Figure 1:**
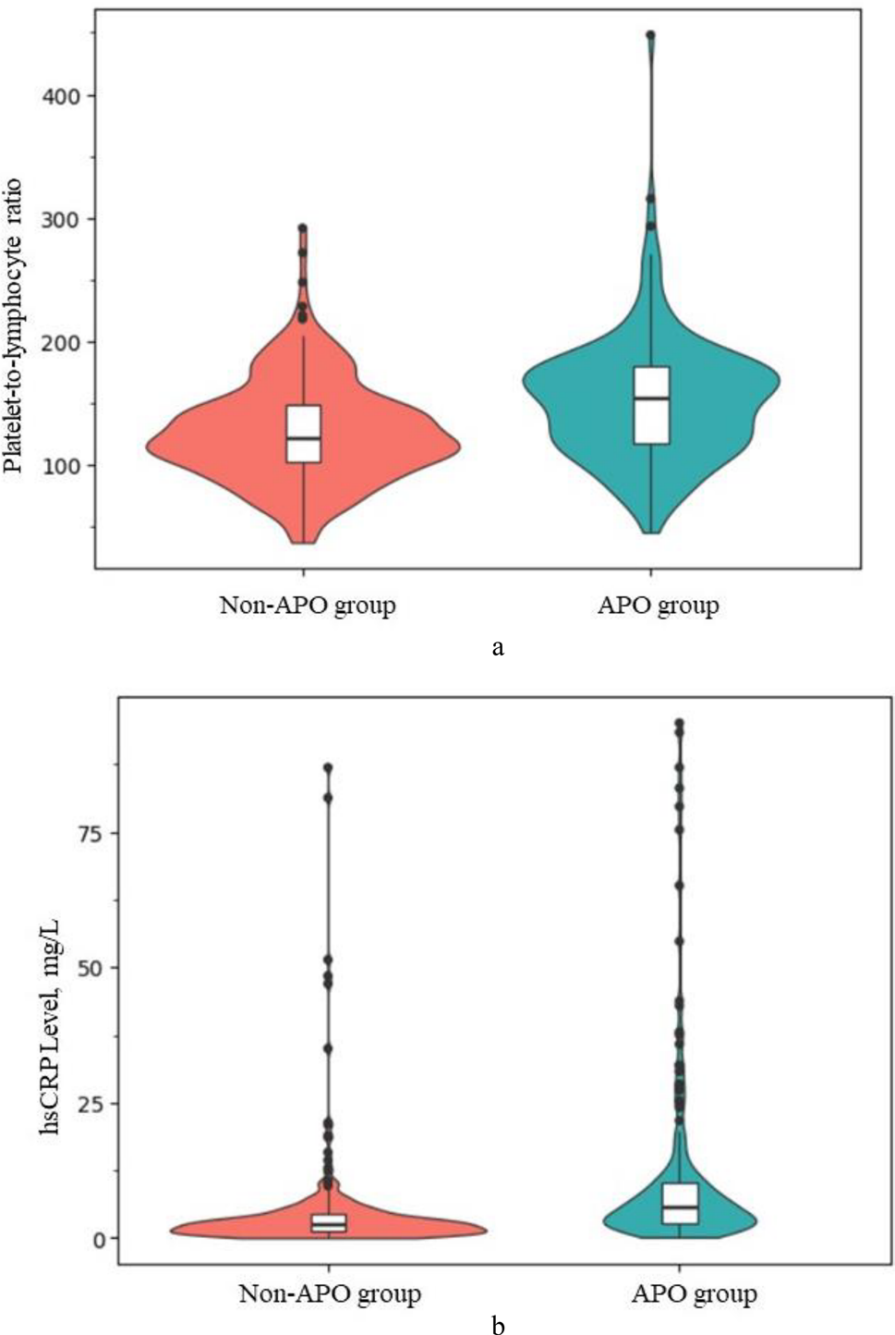
Violinplots comparing PLR and hsCRP between APO group and non-APO group.

APO occurred in 171 of the 406 included participants. Compared with patients without APO, patients with APO were more likely to suffer from preeclampsia. In addition, the values of LYM, PLT, NLR, PLR and hsCRP in the APO group were higher than those in the non-APO group, while the LMR were lower than non-APO group (Fig. 1). Compared with other indicators, there were no statistical differences (Table 1).

**Table 1:** Baseline clinical features of the APO and non-APO groups.

Characteristics	APO group (n = 171)	non-APO group (n = 235)	*P* value
Clinical variables			
Age (years)	32.00(29.00, 33.00)	32.00(30.00,33.00)	0.360
Gestational weeks	31.00(29.00,32.00)	30.00(28.00,32.00)	0.144
BMI	24.67(22.15, 27.32)	24.39(21.43, 27.71)	0.896
Maternal family History of diabetes (n,%)	88(51.46)	123(52.34)	0.861
Hypertension (n,%)	67(39.18)	90(38.30)	0.857
Insulin therapy (n,%)	74(43.27)	94(40.00)	0.508
Preeclampsia (n, %)	36(21.05)	31(13.19)	0.035
Pregnancy weight gain (kg)	14.17(12.99, 15.60)	14.32(12.08,15.30)	0.989
Glu (mmol/l)	4.97(4.35,5.83)	4.96(4.30,5.82)	0.883
Urinary protein (g/24 h)	0.23 (0.14, 0.31)	0.22(0.15, 0.28)	0.315
RBC (× 10^12^/l)	3.79(3.37,4.12)	3.90 (3.39, 4.34)	0.091
Hb (g/l)	119.00 (106.00, 127.00)	121.00 (107.00, 133.00)	0.111
RDW (%)	15.00 (13.30, 16.30)	14.60 (13.40, 16.10)	0.902
WBC (× 10^9^/l)	8.15 (7.02,9.54)	8.46 (7.02, 9.84)	0.498
NEU (× 10^9^/l)	5.93 (5.07, 7.25)	6.08 (4.96, 7.22)	0.827
LYM (× 10^9^/l)	1.40 (1.18, 1.71)	1.56 (1.33, 1.91)	< 0.001
MONO (× 10^9^/l)	0.58 (0.47, 0.70)	0.58 (0.46, 0.71)	0.963
PLT (× 10^9^/l)	213.90 ± 57.13	197.95 ± 49.97	0.003
MPV (fL)	10.70 (10.00, 11.60)	10.90 (10.10, 12.00)	0.108
PDW (fL)	12.50 (11.10, 14.60)	13.10 (11.60, 15.10)	0.080
NLR	4.26 (3.31, 5.39)	3.81 (3.01, 4.92)	0.002
LMR	2.39 (1.97, 3.15)	2.79 (2.25, 3.46)	< 0.001
PLR	153.61 (116.49, 180.14)	121.71 (102.53, 148.39)	< 0.001
hsCRP (mg/l)	5.58 (2.83, 10.28)	2.53 (1.26, 4.62)	< 0.001

Continuous data were presented as mean ± SD or median (interquartile range, IQR) and compared using Student’s independent t-test or Mann–Whitney U test, while categorical data were presented as percentage (%) and compared using Chi-Square test. BMI, Body Mass Index; Glu, glucose; RBC, Red blood cell count; Hb, hemoglobin; RDW, Red blood cell distribution width; WBC, White blood cell count; Neu, Neutrophil count; LYM, lymphocyte count; MONO, monocyte count; PLT, Platelets; MPV, Mean platelet volume; PDW, Platelet distribution width; NLR, Neutrophil–lymphocyte ratio; LMR, Lymphocyte-monocyte ratio; PLR, Platelet-lymphocyte ratio; hsCRP, high-sensitivity C-reactive protein.

### Risk factors for APO in patients with GDM

Univariate and multivariate logistic regression analyses were performed to assess the impact of variables on the prevalence of APO. Variables with *P* < 0.1 in univariate analysis were included in the multivariate model. The results of multivariate logistic regression analysis showed that preeclampsia (OR (95% CI): 1.872 (1.057–3.316), *P* = 0.032), PLR (OR (95% CI): 1.013 (1.006–1.019), *P* < 0.001), and hsCRP levels (1.046 (1.022–1.070), *P* = 0.001) were risk factors for APO in GDM patients (Table 2).

**Table 2:** Univariate and multivariate analyses for the risk factors of APO in patients with GDM.

Variables	OR (95%CI)	*P* value	OR (95%CI)	*P* value
Age	0.981 (0.925–1.040)	0.518		
Gestational weeks	1.028 (0.973–1.087)	0.324		
BMI	1.009 (0.954–1.066)	0.760		
Maternal family history of diabetes	0.965 (0.651–1.432)	0.861		
Hypertension	1.038 (0.693–1.555)	0.857		
Insulin therapy	1.144 (0.767–1.706)	0.508		
Preeclampsia	1.755 (1.036–2.973)	0.037	1.872 (1.057–3.316)	0.032
Pregnancy weight gain	1.008 (0.881–1.153)	0.906		
Glu	0.922 (0.796–1.067)	0.275		0.883
Urinary protein	2.725 (0.804, 9.235)	0.107		
RBC	0.778 (0.568–1.066)	0.119		
Hb	0.991 (0.980–1.001)	0.086	0.990 (0.979–1.002)	0.107
RDW	1.005 (0.923–1.094)	0.913		
WBC	0.977(0.898–1.064)	0.600		
MPV	0.857 (0.731–1.006)	0.059	0.941 (0.746–1.188)	0.611
PDW	0.940 (0.873–1.012)	0.098	1.000 (0.909–1.101)	0.995
NLR	1.173 (1.044–1.317)	0.007	0.971 (0.832–1.133)	0.706
LMR	0.708 (0.568–0.883)	0.002	0.901 (0.681–1.191)	0.463
PLR	1.013 (1.008–1.018)	< 0.001	1.013 (1.006–1.019)	< 0.001
hsCRP	1.048 (1.023–1.073)	< 0.001	1.046 (1.022–1.070)	< 0.001

BMI, Body Mass Index; Glu, glucose; RBC, Red blood cell count; Hb, hemoglobin; RDW, Red blood cell distribution width; WBC, White blood cell count; Neu, Neutrophil count; LYM, lymphocyte count; MONO, monocyte count; PLT, Platelets; MPV, Mean platelet volume; PDW, Platelet distribution width; NLR, Neutrophil–lymphocyte ratio; LMR, Lymphocyte-monocyte ratio; PLR, Platelet-lymphocyte ratio; hsCRP, high-sensitivity C-reactive protein.

### Association of PLR and hsCRP with adverse pregnancy outcomes

To analyze the association between the prevalence of adverse pregnancy outcomes and the PLR or hsCRP level, we divided patients into PLR quartiles and calculated the odds ratios (ORs) of their risk of APO, taking patients in the fourth PLR quartiles as a reference (Table 3). Similarly, Table 4 describes the ORs and 95% CIs of the prevalence of APO for hsCRP level. In the unadjusted Model 1, the PLR and hsCRP level were positively correlated with the prevalence of APO. After age and gestational weeks adjusted in Model 2, the results were similar to those of Model 1. After further controlling for BMI, Maternal family history of diabetes, hypertension, Insulin therapy, Preeclampsia, Pregnancy weight gain, Glu, Urinary protein, RBC, Hb, RDW, WBC, NEU, LYM, MONO, PLT, MPV, PDW, NLR, LMR in Model 3, the association was still statistically significant and changed little. The fully adjusted ORs in Model 3 was 0.238 (95% CI: 0.105–0.539) for those in quartile 1 of PLR (the lowest) versus quartile 4 (the highest) and was 0.093 (95% CI: 0.045–0.191) for those in in quartile 1 of serum hsCRP level(the lowest) versus quartile 4 (the highest) (Tables 3 and 4).

**Table 3:** Association of the prevalence of APO with the PLR.

PLR quartile	n	Ratio range	OR (95%CI)		
			Model 1	Model 2	Model 3
Quartile 4 (high)	101	> 167.83	Reference	Reference	Reference
Quartile 3	101	133.34–167.83	0.412 (0.234–0.725)	0.412 (0.234–0.728)	0.522 (0.272–1.000)
Quartile 2	102	108.34–133.33	0.329 (0.186–0.583)	0.335 (0.189–0.594)	0.362 (0.178–0.736)
Quartile 1 (low)	102	≤ 108.33	0.219 (0.121–0.396)	0.218 (0.120–0.396)	0.238 (0.105–0.539)
*P* for trend			< 0.001	< 0.001	< 0.001

Model 1 no adjustment.

Model 2 adjusted for age and gestational weeks.

Model 3 adjusted for age, gestational weeks, BMI, Maternal family history of diabetes, hypertension, Insulin therapy, Preeclampsia, Pregnancy weight gain, Glu, Urinary protein, RBC, Hb, RDW, WBC, NEU, MONO, MPV, PDW, NLR, LMR, hsCRP.

**Table 4:** Association of the prevalence of APO with the hsCRP.

hsCRP quartile	n	Concentration range, mg/l	OR (95%CI)		
			Model 1	Model 2	Model 3
Quartile 4 (high)	101	> 6.84	Reference	Reference	Reference
Quartile 3	101	3.33–6.83	0.326 (0.183–0.583)	0.326 (0.181–0.587)	0.300 (0.155–0.580)
Quartile 2	102	1.70–3.32	0.221 (0.122–0.399)	0.219 (0.121–0.399)	0.227 (0.118–0.435)
Quartile 1 (low)	102	≤ 1.69	0.110 (0.058–0.208)	0.109 (0.057–0.207)	0.093 (0.045–0.191)
*P* for trend			< 0.001	< 0.001	< 0.001

Model 1 no adjustment.

Model 2 adjusted for age and gestational weeks.

Model 3 adjusted for age, gestational weeks, BMI, Maternal family history of diabetes, hypertension, Insulin therapy, Preeclampsia, Pregnancy weight gain, Glu, Urinary protein, RBC, Hb, RDW, WBC, NEU, LYM, MONO, PLT, MPV, PDW, NLR, LMR, PLR.

### Nonlinear association of PLR and hsCRP with adverse pregnancy outcomes

**Figure 2:**
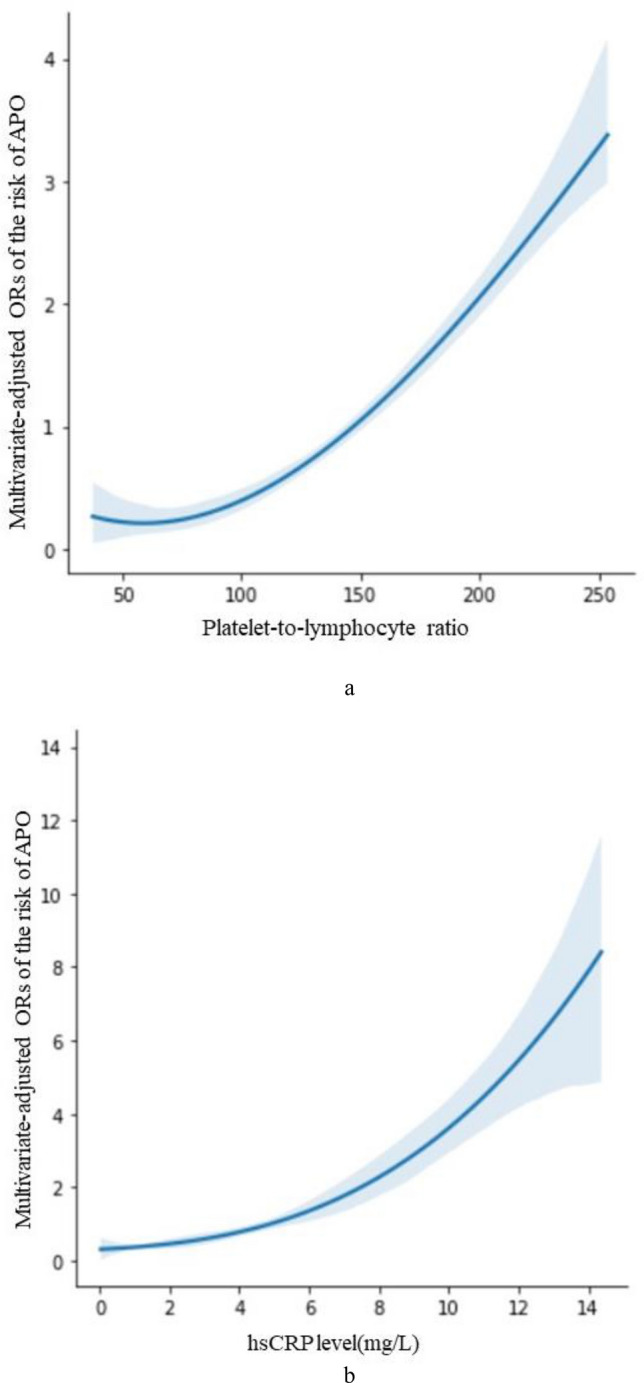
Nonlinear associations between PLR and hsCRP and the risk of APO.

Restricted cubic spline analyses found that after adjusting for all confounding factors included in this study, the risk of APO increases with increased levels of PLR and hsCRP in the third trimester (Fig. 2).

### Diagnostic efficacy of PLR and hsCRP for adverse pregnancy outcomes

**Figure 3:**
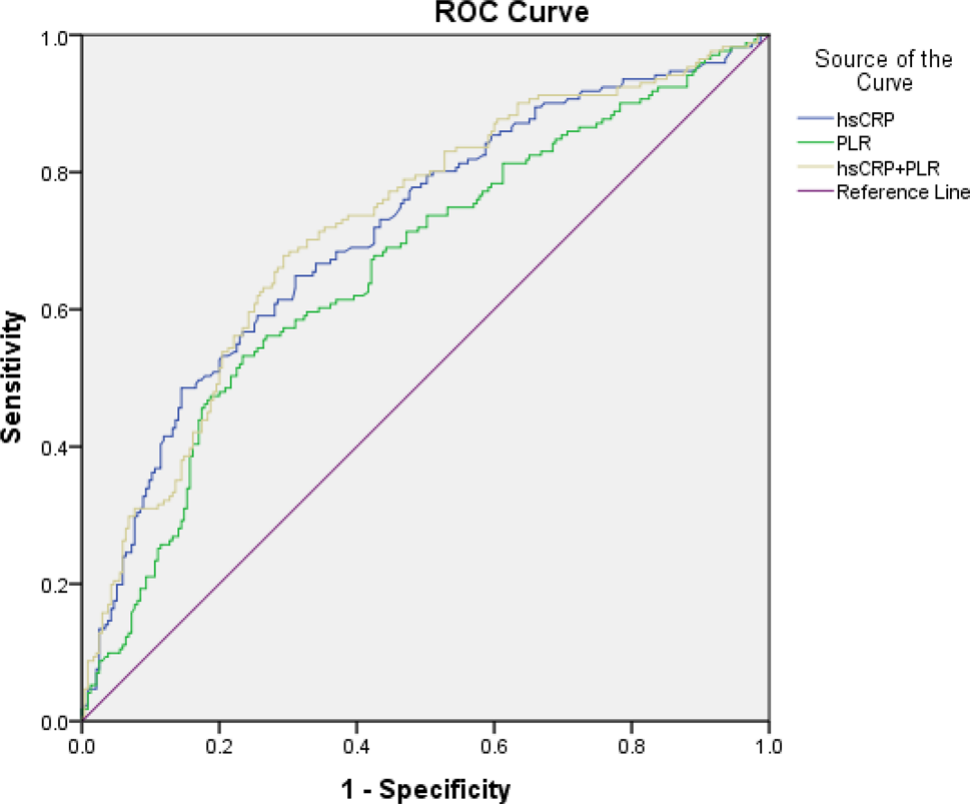
ROC curve of PLR and hsCRP in predicting the occurrence of APO.

As shown in Fig. 3, ROC curve showed that the AUC of hsCRP in predicting the occurrence of APO was 0.714 [95% CI 0.663–0.765], the optimal cut-off value was 3.73 mg/l, the Youden Index was 0.338, the sensitivity was 64.9%, and the specificity was 68.9%. ROC curve showed that the AUC of PLR in predicting the occurrence of APO was 0.661 [95% CI 0.607–0.715], the optimal cut-off value was 149.15, the Youden Index was 0.298, the sensitivity was 53.2%, and the specificity was 76.6%. Furthermore, according to logistic regression analysis, the AUC for the combined predicted probability of hsCRP and PLR was 0.722 [95% CI 0.672–0.772] (Fig. 3).

## Discussion

This study is the first to identify PLR and hsCRP levels as potential risk factors for APO in pregnant women with GDM. This correlation persisted after adjusting for confounders.

GDM is one of the most common complications during pregnancy, with an incidence of 5–6% to 15–20% in different parts of the world, and the incidence is still increasing, mainly related to obesity, sedentary lifestyle and increasing pregnancy age^25–28^. GDM not only increases the risk of APO such as preeclampsia, polyhydramnios, macrosomia, and fetal distress, but also increases the incidence of long-term cardiovascular disease in both mother and child generations^8–10^. Early intervention in women with GDM can improve the occurrence of adverse maternal and fetal outcomes. Therefore, identifying high-risk patients with APO and intervening them are of great significance for the prevention and treatment of APO in pregnant women with GDM.

Numerous studies have demonstrated that PLR, and hsCRP are potential inflammatory biomarkers for rheumatoid arthritis, endocrine disease, cardiovascular disease, and tumors^29–33^. In addition, there is mounting evidence connecting them to the progression of GDM and its complications. Wang et al.^34^ found a significant increase in PLR among pregnant women in the GDM group through analysis of blood indicators in 1440 early pregnant women. Moreover, their research has shown that PLR in early pregnancy is an independent risk factor for GDM. In a similar study, Fashami et al.^35^ found a significant increase in PLT values and PLR in the GDM group during the second trimester of pregnancy. The findings of the Maged et al. study have shown that hsCRP is an important early predictor of GDM^36^. A prospective study of women with gestational age less than 10 weeks confirmed that women with the highest serum concentration of CRP were three times more likely to develop GDM than women with the lowest serum concentration of CRP^37^. In addition, the results of a study conducted by Ozugz et al.^38^ showed that during the 1-year follow-up evaluation, the hsCRP in the GDM group was significantly higher than that in the control group, and the carotid intima-media thickness was significantly greater than that in the control group. They defined GDM as a transient metabolic syndrome and a subclinical inflammatory state associated with elevated hsCRP levels. Previous studies mainly focused on the correlation between PLR and hsCRP and the prevalence of GDM, but there were few studies on their association with APO in pregnant women with GDM. This study analyzed the blood parameters of 406 pregnant women with GDM in the third trimester of pregnancy, and found that there was a significant correlation between the levels of PLR and hsCRP and the occurrence of APO in GDM pregnant women.

The reasons for the prevalence of APO are not fully understood, and previous studies have found that inflammation is associated with APO in pregnant women with GDM^13,39–41^. Both PLR and hsCRP are commonly used indicators to reflect the inflammatory state of the body. Platelets are rich in pro-inflammatory agents and can release highly active microparticles, which are related to the inflammatory state of the body. In vitro and in vivo studies have elucidated the origin of activated circulating platelets, which display numerous membrane receptors and release a variety of bioactive substances from their granules that can modulate cellular interactions and contribute to immune, inflammatory, and thrombotic diseases^42–44^. P-selectin-mediated interaction of platelets with T lymphocytes reduces lymphocyte proliferation and modulates the release of inflammatory factors, leading to activation of the inflammatory response^45,46^. As a sensitive indicator of non-specific inflammation, hsCRP mainly inhibits the activity of insulin receptor tyrosine kinase and phosphorylates insulin receptor substrates, resulting in the disorder of the body's material metabolism, thereby aggravating IR^47–50^.

This study has several limitations. First, due to the small sample size, other risk factors for APO were not stratified. Second, although we found elevated levels of PLR and hsCRP in GDM pregnant women in the third-trimester APO group, we were unable to collect any data on PLR and hsCRP levels in the disease during the first and second trimesters. Blood samples obtained longitudinally during pregnancy may add more comprehensive data to understand the value of PLR and hsCRP levels during disease development.

## Conclusion

In short, the levels of PLR and hsCRP in the third trimester are related to the occurrence of APO in women with GDM, and high levels of PLR and hsCRP may indicate the higher risk of the occurrence of APO. If the association and related mechanisms between PLR and hsCRP with APO in patients with GDM are confirmed in future research, potential interventions to improve maternal and infant outcomes can be expected.

## Data Availability

The datasets generated during and analysed during the current study are not publicly available due to privacy or ethical restrictions but are available from the corresponding author on reasonable request.
